# Graft Survivorship After Anterior Cruciate Ligament Reconstruction Based on Tibial Slope

**DOI:** 10.1177/03635465211049234

**Published:** 2021-10-21

**Authors:** Clemens Gwinner, Milan Janosec, Guido Wierer, Michael Wagner, Andreas Weiler

**Affiliations:** † Center for Musculoskeletal Surgery, Charité-University Medicine Berlin, Germany; ‡ Department of Orthopaedics and Traumatology, Paracelsus Medical University, Salzburg, Austria; § Research Unit for Orthopaedic Sports Medicine and Injury Prevention, Private University for Health Sciences, Medical Informatics and Technology, Hall, Austria; ‖ Sporthopaedicum, Berlin, Germany; Investigation performed at the Charité-University Medicine, Berlin, Germany, and the Sporthopaedicum, Berlin, Germany

**Keywords:** anterior cruciate ligament, ligament reconstruction, tibial slope, revision surgery, repeated graft insufficiency

## Abstract

**Background:**

Increased tibial slope (TS) is believed to be a risk factor for anterior cruciate ligament (ACL) tears. Increased TS may also promote graft insufficiency after ACL reconstruction.

**Purpose:**

To delineate the relationship between TS and single as well as multiple graft insufficiencies after ACL reconstruction.

**Study Design:**

Cohort study; Level of evidence 3.

**Methods:**

We retrospectively identified 519 patients who had sustained ACL graft insufficiency after primary or revision ACL reconstruction (1 graft insufficiency, group A; 2 graft insufficiencies, group B; and ≥3 graft insufficiencies, group C). In addition, a subgroup analysis was conducted in 63 patients who received all surgical interventions by 2 specialized high-volume, single-center ACL surgeons. TS was measured by an observer with >10 years of training using lateral knee radiographs, and intrarater reliability was performed. Multiple logistic and univariate Cox regression was used to assess the contribution of covariates (TS, age, sex, and bilateral ACL injury) on repeated graft insufficiency and graft survival.

**Results:**

The study included 347 patients, 119 female and 228 male, who were 24 ± 9 years of age at their first surgery (group A, n = 260; group B, n = 62; group C, n = 25). Mean TS was 9.8°± 2.7° (range, 3°-18°). TS produced the highest adjusted odds ratio (1.73) of all covariates for repeated graft insufficiency. A significant correlation was found between TS and the number of graft insufficiencies (*r* = 0.48; *P* < .0001). TS was significantly lower in group A (9.0°± 2.3°) compared with group B (12.1°± 2.5°; *P* < .0001) and group C (12.0°± 2.6°; *P* < .0001). A significant correlation was seen between the TS and age at index ACL tear (*r* = −0.12; *P* = .02) as well as time to graft insufficiency (*r* = −0.12; *P* = .02). A TS ≥12° had an odds ratio of 11.6 for repeated ACL graft insufficiency.

**Conclusion:**

The current results indicate that patients with a markedly increased TS were at risk of early and repeated graft insufficiency after ACL reconstruction. Because the TS is rarely accounted for in primary and revision ACLR, isolated soft tissue procedures only incompletely address recurrent graft insufficiency in this subset of patients.

The functional importance of the anterior cruciate ligament (ACL) has been widely acknowledged. After injury, reconstruction of the ACL is deemed necessary to restore native knee kinematics close to the physiological state and to allow athletes to return to high-level pivoting and cutting sports.

Surgical techniques for ACL reconstruction (ACLR) continue to evolve and to improve clinical results. Even though early results appear to be satisfactory, outcome parameters in the long term seem to deteriorate and the true incidence of graft insufficiency after ACLR is likely underreported.^22^ These graft insufficiencies are worrisome, because revision ACLR is associated with worse outcomes compared with primary ACLR.^30,32,33^

Even though the contributions of peripheral soft tissue restraints on sagittal knee laxity have been validated extensively, the importance of the bony geometry of the underlying tibial plateau is less well understood. However, a growing body of evidence has provided a refined view on the effect of the posterior inclination of the tibial plateau—namely, the tibial slope (TS)—on anteroposterior knee laxity and loading of the cruciate ligaments.^14,24,28,31^ An emerging consensus indicates that from a biomechanical view, an increased TS facilitates an anterior translation of the tibia relative to the femur.^1,7,8,10,23^ Clinical studies have echoed these results, reporting an association between an increased TS and graft insufficiency after ACLR.^3,12,15,22,29^ However, there is a considerable paucity in the current literature regarding the TS in large revision ACL cohorts, and its effect on singular and multiple graft insufficiencies has not been delineated.

Of note, the susceptibility for graft insufficiency is dependent not only on anatomic conditions but also, and to a greater degree, on age, acquired injuries, graft tissue, graft position, treatment of additional peripheral instabilities, and postoperative rehabilitation.^18^ The TS is rarely accounted for in surgical decision making in revision ACLR, thus potentially representing a nonmodifiable risk factor for repeated graft insufficiency.

The aim of the current study was to evaluate the TS in patients with single and multiple graft insufficiencies after ACLR. It was hypothesized that the TS would be significantly higher in patients with multiple graft insufficiencies compared with those with a single graft insufficiency.

## Methods

A total of 519 patients who underwent revision ACLR between 2012 and 2018 due to graft insufficiency were retrospectively identified from a single-center, 2-surgeon series (A.W., M.W.). Inclusion criteria consisted of single or multiple ACL graft insufficiencies after ACLR, confirmed by magnetic resonance imaging (MRI) and/or clinical as well as arthroscopic assessment.

Patients with concomitant injuries of the posterior cruciate ligament (PCL), combined lower extremity fractures, or previous osteotomy of the ipsilateral knee were excluded. In addition, we excluded patients whose radiographs did not include a true lateral view of the tibia as determined by proper superimposition of the femoral condyles. Patients with poor-quality or nondigital radiographs were not enrolled in order to decrease the number of confounding variables.

Anthropometric and demographic measures including age and sex were documented. Additionally, for each patient, we reported the number of graft insufficiencies (1 graft insufficiency, group A; 2 graft insufficiencies, group B; ≥3 graft insufficiencies, group C), graft choice, the patient’s age at first ACL injury or ACLR, and the subsequent time interval (months) between ACLR and graft insufficiency. This information was also collected for a subgroup of patients who underwent both the primary ACLR and subsequent revision surgeries by either of the 2 specialized high-volume ACL surgeons (A.W., M.W.).

The study protocol was approved by our institutional ethics committee (EA2/016/21). The study was carried out in accordance with the Declaration of Helsinki, and all participants provided informed consent.

### Radiographic Assessment

Lateral knee radiographs of the affected knee were used for radiographic assessment. An orthopaedic surgeon (C.G.) with >10 years of experience in radiographic measurements evaluated the TS using a picture archiving and communication system workstation (Centricity RIS-I 4.2 Plus; GE Healthcare). The observer was blinded for all other parameters. To account for reading inaccuracy, 50 patients were randomly selected and measured again at 6 months after the initial assessment to establish intrarater reliability.

**Figure 1.**
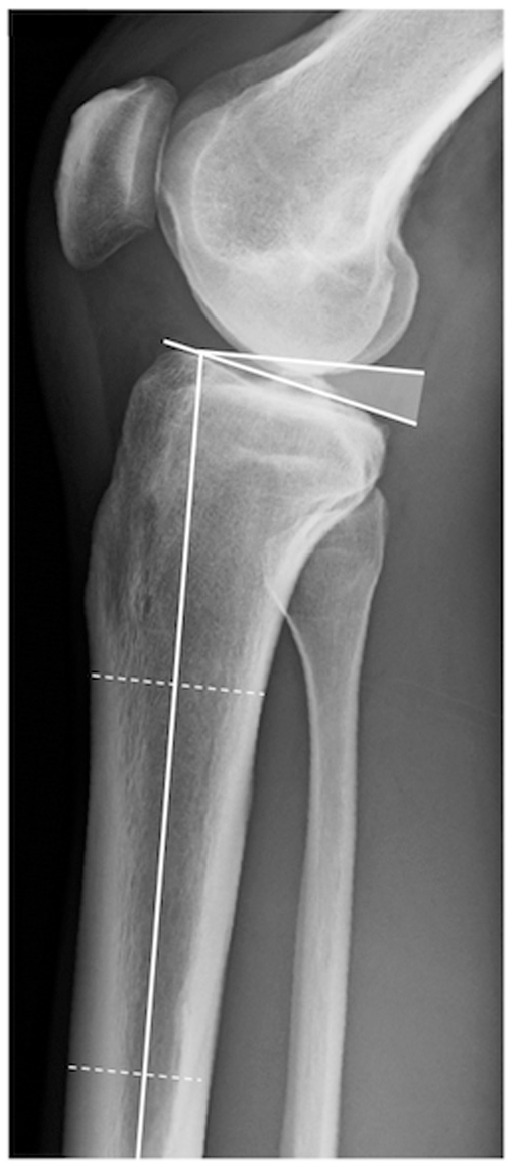
Measurement of the tibial slope (TS). The TS is defined as the angle (shaded) between the tangent line of the medial tibial plateau and a line perpendicular to the tibial shaft axis.

The TS is regarded as the angle between the posterior inclination of the tibial plateau and a line perpendicular to the tibial shaft axis. The tibial shaft axis was determined according to Dejour and Bonnin^8^ using 2 midpoints between the anterior and posterior tibial cortex: one at 5 cm below the tibial tuberosity and the other at 15 cm below the tibial joint line. Subsequently, the TS was determined between the tangent line to the medial tibial plateau, which is commonly superimposed on lateral radiographs, and a line 90° to the diaphyseal axis. Although the best available radiographs were chosen, not all images included enough tibial shaft to determine the width of the shaft at a distance of 15 cm distal to the joint line. In these instances, the most distal width (if ≥12 cm)^13^ was measured based on the aforementioned conditions (Figure 1).

### Statistical Analysis

Statistical analysis was performed using Prism Version 6 (GraphPad Software). Continuous data are expressed as mean ± SD or median according to their distribution. D’Agostino and Pearson omnibus normality tests were used to test Gaussian distribution. Parametric data were compared with a *t* test, and nonparametric data were tested by a Mann-Whitney test. Either Pearson or Spearman correlations were used for the statistical association between 2 variables, according to their normality test. Multiple logistic regression was performed to assess the relative contribution of age, sex, bilateral ACL tears, and TS on the number of graft insufficiencies. The Pearson chi-square test was used to determine statistically significant differences between categorical variables. Survival curves were compared with univariate Cox regression. The intraclass correlation coefficient (ICC) was used to evaluate intrarater reliability. Notably, values of 0.5 through 0.6 are regarded as moderate agreement, whereas 0.7 through 0.8 indicate a strong agreement. *P* < .05 was considered to be statistically significant.

## Results

**Figure 2.**
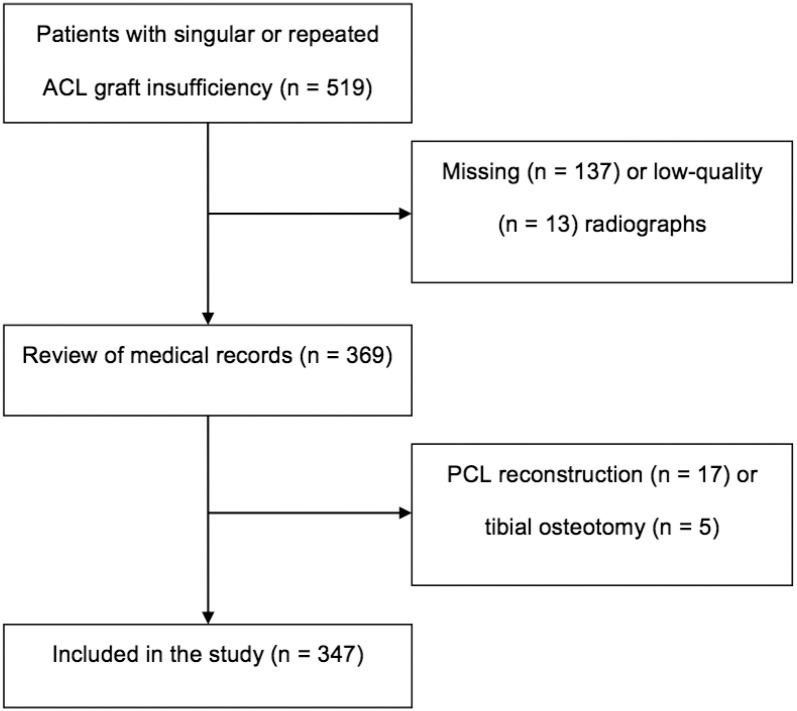
Flowchart depicting the selection of study participants. ACL, anterior cruciate ligament; PCL posterior cruciate ligament.

Of the 519 patients who were identified after sustaining ACL graft insufficiency, patients were excluded due to missing (n = 137) or low-quality (n = 13) radiographs, additional PCL reconstruction (n = 17), or tibial osteotomy (n = 5). Consequently, 347 patients (119 female, 228 male) were enrolled in the present study (Figure 2). The mean age of the patients at first ACL rupture was 24 ± 9 years. The ICC was 0.86, indicating a high intrarater agreement in terms of TS measurement.

According to the number of graft insufficiencies, 260 patients were enrolled with 1 graft insufficiency (group A), 62 patients with 2 graft insufficiencies (group B), and 25 patients with ≥3 graft insufficiencies (group C; 22 patients with 3 graft insufficiencies and 3 patients with 4 graft insufficiencies). Patient characteristics among the subgroups are described in Table 1.

**Table 1:** Patient Characteristics of the Subgroups^*a*^

	Group A	Group B	Group C
No. of patients	260	62	25
Tibial slope, deg, mean ± SD	9 ± 2	12 + 3	12 + 3
Age at first surgery, y, mean ± SD	26 ± 9	21 ± 7	21 ± 6
Female/male, n	81/179	29/33	9/16
Bilateral ACL tears, n (%)	31 (12)	11 (17)	6 (24)

a ACL, anterior cruciate ligament; group A, patients with 1 graft insufficiency; group B, patients with 2 graft insufficiencies; group C, patients with ≥3 graft insufficiencies.

Multiple logistic regression was performed to assess the relative contribution of age, sex, bilateral ACL tears, and TS on the number of graft insufficiencies. The results are shown in Table 2. Repeated graft insufficiency was associated with TS (odds ratio [OR], 1.73; 95% CI, 1.5-1.99) and age (OR, 0.99; 95% CI, 0.99-1) and, to a lesser extent, with female sex (OR, 0.47; 95% CI, 0.25-0.88) but not with the presence of bilateral ACL tears.

**Table 2:** Results of Regression Analysis: Relationship Between Multiple Graft Insufficiencies and Age, Sex, Bilateral ACL Tears, and Tibial Slope^*a*^

Factor	Adjusted OR	95% CI	*P* Value
Age	0.99	0.99-1	.001
Female sex	0.47	0.25-0.88	.018
Bilateral ACL tears	0.49	0.22-1.07	.074
Tibial slope	1.73	1.5-1.99	<.0001

a ACL, anterior cruciate ligament; OR, odds ratio.

### Analysis of the Tibial Slope

**Figure 3.**
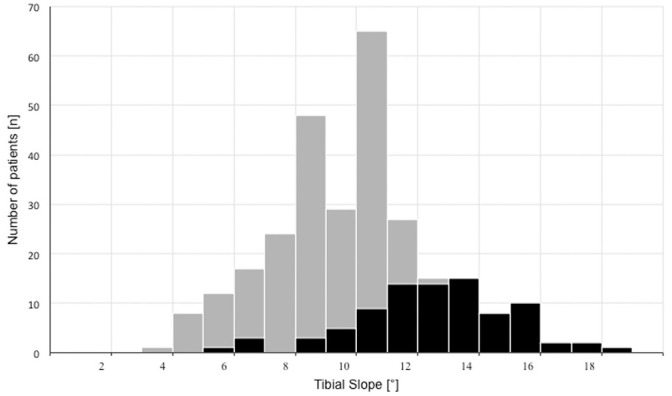
Distribution of the tibial slope in patients with single (gray) and multiple (black) graft insufficiencies.

The mean TS was 9.8°± 2.7° (range, 3°-18°) (Figure 3). A significant correlation was seen between the TS and the stage of graft insufficiency (Spearman correlation, *r* = 0.48; *P* < .0001).

**Figure 4.**
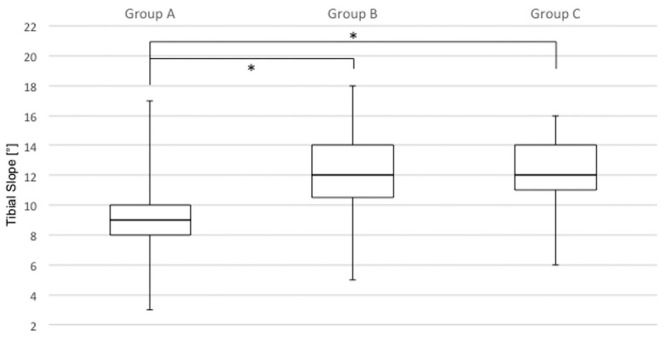
Boxplot comparing the tibial slope between patients with 1 graft insufficiency (group A), 2 graft insufficiencies (group B), and ≥3 graft insufficiencies (group C). The tibial slope was significantly different between groups A and B as well as between groups A and C. *Statistically significant.

More specifically, a significant difference in TS was seen between groups A and B (9.0°± 2.3° vs 12.1°± 2.5°, respectively; *P* < .0001) as well as between groups A and C (9.0°± 2.3° vs 12.0°± 2.6°, respectively; *P* < .0001). No significant difference was seen between groups B and C (12.1°± 2.5° vs 12.0°± 2.6°, respectively) (Figure 4).

Using a cutoff of ≥12°, as proposed by Webb et al,^29^ we found that the rate of sustaining repeated graft insufficiencies was significantly higher in patients exceeding this cut-off (64%) compared with those who had a TS <12° (13%) (*P* < .0001). More specifically, a TS ≥12° produced an odds ratio of 11.6 for repeated ACL graft insufficiency.

### Anthropometric Analysis

**Figure 5.**
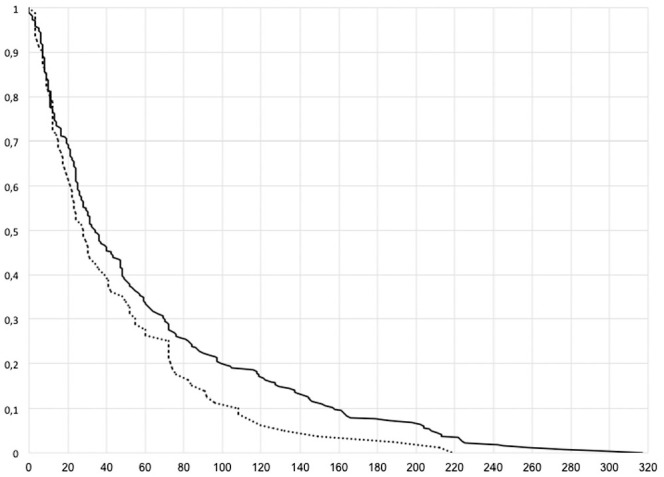
Kaplan Meier curve showing the survivorship (months) of anterior cruciate ligament grafts in patients with a tibial slope ≥12° (dotted line) and <12° (black line).

The mean age of the patients at first ACL tear was 24 ± 9 years. In addition to the multiple logistic regression analysis, Spearman correlation revealed a weak yet significant correlation between age at ACL tear and TS (*r* = −0.12; *P* = .02). The interval between ACLR and first graft insufficiency was 57 ± 61 months. As well, we found a significant correlation between the TS and the time to graft insufficiency (*r* = −0.12; *P* = .02). Graft survivorship after ACLR was significantly impaired in patients with TS ≥12° (Figure 5).

The interval between revision ACLR and second graft insufficiency was 37 ± 35 months. This is significantly shorter than the time between ACLR and first graft insufficiency (57 ± 61 months; *P* = .005).

We compared the TS in female (n = 119) versus male (n = 228) patients. No significant difference was seen regarding the TS in both cohorts (9.7°± 2.7° vs 9.8°± 2.7°, respectively). A total of 49 male (22%) and 38 female (32%) patients sustained repeated graft insufficiency, which resulted in a weak, yet significant, difference (*P* = .046). This is in line with the regression analysis, which showed a weak association between female patients and repeated graft insufficiency.

A total of 48 patients sustained bilateral ACL injuries. However, we found no significant difference in the TS between patients with unilateral and bilateral ACL injuries (9.7°± 2.7° vs 10.0°± 2.9°, respectively).

### Graft Choice

Ipsilateral hamstring tendons (n = 236) were predominantly used during index ACLR, followed by ipsilateral patellar tendons (n = 74) and quadriceps tendons (n = 5). In 32 patients, the primary graft choice was unknown. In the hamstring tendon group, 56 patients (24%) had >1 graft insufficiency. In the patellar tendon group, 21 patients (28%) had >1 graft insufficiency. However, this difference did not reach statistical difference.

For revision ACLR, autologous hamstring tendons of the ipsilateral (if not previously used) or contralateral side were used in 268 patients. Quadriceps tendons were used in 48 patients and patellar tendons in 18 patients. The graft was unknown in 13 patients. In this ACL revision setting, 48 (18%) of the hamstring tendon grafts, 16 (33%) of the quadriceps tendon grafts, and 18 (100%) of the patellar tendon grafts failed. In revision ACLR, hamstring tendon grafts showed a significantly lower rate for repeated insufficiency compared with quadriceps tendon grafts (*P* = .024) and patellar tendon grafts (*P* < .0001). However, the TS was significantly higher in patients with patellar tendon grafts (11.3°± 3.1°) than in patients with hamstring tendon grafts (9.6°± 2.6°; *P* = .008) and quadriceps tendon grafts (9.8°± 2.7°; *P* = .046). The TS in patients receiving hamstring tendon grafts and quadriceps tendon grafts was not significantly different.

### Single Center, 2-Surgeon Series

**Figure 6.**
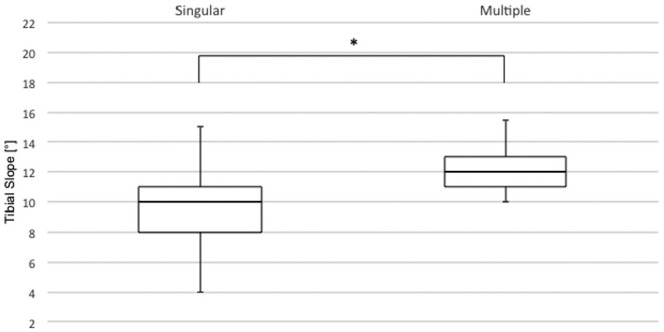
Boxplot showing that the tibial slope in patients with a single graft insufficiency was significantly different than for those with multiple graft insufficiencies. *P < .05.

A total of 63 patients received all ACL surgeries—including primary ACLR—by either of the 2 specialized high-volume ACL surgeons. The TS was significantly different in patients who sustained a single graft insufficiency (n = 57) compared with those with multiple graft insufficiencies (n = 6) (9.4°± 2.1° vs 12.6°± 1.7°, respectively; *P* < .001) (Figure 6).

Because the graft choice for primary and revision ACLR was predominantly ipsilateral or contralateral hamstring tendons, and bone-tendon-bone or quadriceps tendons were sparsely used, no further analysis on the graft choice was conducted in this cohort.

## Discussion

The results of the current study indicate an association between an increased TS and the incidence of repeated graft insufficiency after ACLR. More specifically, knees with a radiographic TS ≥12° had a 64% incidence of repeated graft insufficiency, which produced an odds ratio of 11.6 for repeated ACL graft insufficiency. Notably, the TS also significantly correlated with a younger patient age at first ACL injury and further correlated with a shorter time between primary surgery and first ACL graft insufficiency. Consequently, patients with a markedly increased TS are at risk of sustaining an ACL tear early in their lives and are at further risk of an impaired survival rate as well as survival time of their ACL graft.

Despite recent advances in primary and revision ACL surgery, a considerable number of patients do not recover from revision ACLR, clinical results remain poor compared with primary procedures, and the risk of repeated graft insufficiency is considerably higher.^4,11,20^ Although proper choice of graft tissue, graft position, tensioning, treatment of additional peripheral instabilities, and postoperative rehabilitation continue to be the mainstays for good clinical outcomes, the influence of the underlying osseous geometry of the operated knee on clinical outcome has long been underestimated. Yet, the TS may be considered a common, nonmodifiable risk factor for successive failures.^25^ Numerous studies have identified an increased medial as well lateral TS to be a risk factor for both primary ACL injury and ACL graft insufficiency.^¶^ Of note, these findings contrast with a recent case-control study that delineated the TS in 317 revisions. Cooper et al^5^ failed to observe an association between graft insufficiency and medial as well as lateral TS as measured on MRI. Interestingly, the authors found twice as many graft insufficiencies with a TS >12° than below this threshold. However, this result did not reach statistical significance after correction for multiplicity. The conflicting results between Cooper’s study and our findings might be attributable to the fact that Cooper et al recruited their cohort from a large database in which the number of involved surgeons, their degree of specialization, and surgical volume were unknown. Notably, the TS of the single-center patients in the current study, who were treated in the same surgical fashion by 2 highly specialized ACL surgeons, was significantly higher in patients with multiple graft insufficiencies than in those with a single graft insufficiency.

Webb et al^29^ demonstrated that a TS >12° on lateral radiographs may lead to a 5-fold increase in odds of graft insufficiency after primary ACLR. These results have been echoed by recent studies^19,22^ and are in line with the results of the current study using an ACL revision cohort. As an implication of this, isolated soft tissue procedures may only incompletely address anterior stability in this subset of patients.

Our findings clearly indicate that a high TS significantly correlates with the number of repeated graft insufficiencies and has a detrimental effect on graft survivorship.

A few limitations of this study should be noted. Because we did not account for clinical endpoints, the clinical applicability in regard to patient-reported outcomes or resumption of sporting activities may be limited. Furthermore, the current cohort included only patients with symptomatic graft insufficiency who were undergoing revision ACLR. Because we did not include an asymptomatic ACLR control group, results may be only partially applicable to patients undergoing primary ACLR. Additionally, because most of the patients underwent their index ACLR in other institutions, data such as tunnel positioning, associated injuries, rehabilitation, and return to activity were missing. Thus, potential differences among subgroups cannot be delineated. Of note, the results of the single-center patients who received a standardized treatment and rehabilitation confirm the results of the overall cohort. However, some potentially pertinent factors, such as activity level, could not be considered. Another drawback is that we measured the TS on a standard radiograph, thus potentially ignoring a possible asymmetry between the medial and lateral TS.^9^ Even though the TS appears to be smaller if assessed on MRI, radiographs have been determined to provide an accurate and objective quantification of the TS as well as a high correlation with MRI.^13,16^ In addition, the clinical use of radiography might be greater than the use of MRI assessment, which is more time-consuming and requires specialized software.^13^ Finally, this study does not indicate whether reducing the TS can change the odds of a subsequent graft failure.

## Conclusion

In this study of 347 patients with ACL graft insufficiencies, we showed that patients with a markedly increased TS were at risk of early and repeated graft insufficiency after ACLR. This was particularly distinct in patients with a TS >12°, which produced an odds ratio of 11.6 for repeated ACL graft insufficiency. Consequently, measurement of the TS should be an integral part of failure analysis in cases of ACL graft failure. Future studies need to identify a threshold of the TS that predicts recurrent graft insufficiency after isolated soft tissue revision ACLR.

## Footnotes

¶ References 2,3,6,14,17,21,26,27,29

## References

[1] Agneskirchner JD Hurschler C Stukenborg-Colsman C Imhoff AB Lobenhoffer P . Effect of high tibial flexion osteotomy on cartilage pressure and joint kinematics: a biomechanical study in human cadaveric knees. Winner of the AGA-DonJoy Award 2004. Arch Orthop Trauma Surg. 2004;124:575-584. 15480717 10.1007/s00402-004-0728-8

[2] Bayer S Meredith SJ Wilson K , et al. Knee morphological risk factors for anterior cruciate ligament injury: a systematic review. J Bone Joint Surg Am. 2020;102:703-718. 31977822 10.2106/JBJS.19.00535

[3] Christensen JJ Krych AJ Engasser WM Vanhees MK Collins MS Dahm DL . Lateral tibial posterior slope is increased in patients with early graft failure after anterior cruciate ligament reconstruction. Am J Sports Med. 2015;43:2510-2514. 26320223 10.1177/0363546515597664

[4] Colombet P . Knee laxity control in revision anterior cruciate ligament reconstruction versus anterior cruciate ligament reconstruction and lateral tenodesis: clinical assessment using computer-assisted navigation. Am J Sports Med. 2011;39:1248-1254. 21335352 10.1177/0363546510395462

[5] Cooper JD Wang W Prentice HA Funahashi TT Maletis GB . The association between tibial slope and revision anterior cruciate ligament reconstruction in patients ≤21 years old: a matched case-control study including 317 revisions. Am J Sports Med. 2019;47:3330-3338. 31634002 10.1177/0363546519878436

[6] Dare DM Fabricant PD McCarthy MM , et al. Increased lateral tibial slope is a risk factor for pediatric anterior cruciate ligament injury: an MRI-based case-control study of 152 patients. Am J Sports Med. 2015;43:1632-1639. 26129958 10.1177/0363546515579182

[7] Dejour D Pungitore M Valluy J Nover L Saffarini M Demey G . Preoperative laxity in ACL-deficient knees increases with posterior tibial slope and medial meniscal tears. Knee Surg Sports Traumatol Arthrosc. 2019;27:564-572. 30269166 10.1007/s00167-018-5180-3

[8] Dejour H Bonnin M . Tibial translation after anterior cruciate ligament rupture: two radiological tests compared. J Bone Joint Surg Br. 1994;76:745-749. 8083263

[9] Faschingbauer M Sgroi M Juchems M Reichel H Kappe T . Can the tibial slope be measured on lateral knee radiographs? Knee Surg Sports Traumatol Arthrosc. 2014;22:3163-3167. 24482216 10.1007/s00167-014-2864-1

[10] Giffin JR Vogrin TM Zantop T Woo SL Harner CD . Effects of increasing tibial slope on the biomechanics of the knee. Am J Sports Med. 2004;32:376-382. 14977661 10.1177/0363546503258880

[11] Gifstad T Drogset JO Viset A Grøntvedt T Hortemo GS . Inferior results after revision ACL reconstructions: a comparison with primary ACL reconstructions. Knee Surg Sports Traumatol Arthrosc. 2013;21:2011-2018. 23238924 10.1007/s00167-012-2336-4

[12] Grassi A Signorelli C Urrizola F , et al. Patients with failed anterior cruciate ligament reconstruction have an increased posterior lateral tibial plateau slope: a case-controlled study. Arthroscopy. 2019;35:1172-1182. 30878331 10.1016/j.arthro.2018.11.049

[13] Gwinner C Fuchs M Sentuerk U , et al. Assessment of the tibial slope is highly dependent on the type and accuracy of the preceding acquisition. Arch Orthop Trauma Surg. 2019;139:1691-1697. 31104087 10.1007/s00402-019-03201-y

[14] Hashemi J Chandrashekar N Mansouri H , et al. Shallow medial tibial plateau and steep medial and lateral tibial slopes new risk factors for anterior cruciate ligament injuries. Am J Sports Med. 2010;38:54-62. 19846692 10.1177/0363546509349055

[15] Herman BV Giffin JR . High tibial osteotomy in the ACL-deficient knee with medial compartment osteoarthritis. J Orthop Traumatol. 2016;17:277-285. 27358200 10.1007/s10195-016-0413-z PMC4999379

[16] Hudek R Schmutz S Regenfelder F Fuchs B Koch PP . Novel measurement technique of the tibial slope on conventional MRI. Clin Orthop Relat Res. 2009;467:2066-2072. 19190973 10.1007/s11999-009-0711-3 PMC2706341

[17] Jaecker V Drouven S Naendrup JH Kanakamedala AC Pfeiffer T Shafizadeh S . Increased medial and lateral tibial posterior slopes are independent risk factors for graft failure following ACL reconstruction. Arch Orthop Trauma Surg. 2018;138:1423-1431. 29808437 10.1007/s00402-018-2968-z

[18] Kamath GV Redfern JC Greis PE Burks RT . Revision anterior cruciate ligament reconstruction. Am J Sports Med. 2011;39: 199-217. 20709943 10.1177/0363546510370929

[19] Lee CC Youm YS Cho SD , et al. Does posterior tibial slope affect graft rupture following anterior cruciate ligament reconstruction? Arthroscopy. 2018;34:2152-2155. 29530354 10.1016/j.arthro.2018.01.058

[20] Lind M Lund B Faunø P Said S Miller LL Christiansen SE . Medium to long-term follow-up after ACL revision. Knee Surg Sports Traumatol Arthrosc. 2012;20:166-172. 21800165 10.1007/s00167-011-1629-3

[21] Mitchell JJ Cinque ME Dornan GJ , et al. Primary versus revision anterior cruciate ligament reconstruction: patient demographics, radiographic findings, and associated lesions. Arthroscopy. 2018;34:695-703. 29225019 10.1016/j.arthro.2017.08.305

[22] Salmon LJ Heath E Akrawi H Roe JP Linklater J Pinczewski LA . 20-year outcomes of anterior cruciate ligament reconstruction with hamstring tendon autograft: the catastrophic effect of age and posterior tibial slope. Am J Sports Med. 2018;46:531-543. 29244525 10.1177/0363546517741497

[23] Shelburne KB Kim HJ Sterett WI Pandy MG . Effect of posterior tibial slope on knee biomechanics during functional activity. J Orthop Res. 2011;29:223-231. 20857489 10.1002/jor.21242

[24] Shultz SJ Schmitz RJ . Tibial plateau geometry influences lower extremity biomechanics during landing. Am J Sports Med. 2012;40:2029-2036. 22837428 10.1177/0363546512453295 PMC12329839

[25] Sonnery-Cottet B Archbold P Cucurulo T , et al. The influence of the tibial slope and the size of the intercondylar notch on rupture of the anterior cruciate ligament. J Bone Joint Surg Br. 2011;93:1475-1478. 22058297 10.1302/0301-620X.93B11.26905

[26] Sturnick DR Vacek PM DeSarno MJ , et al. Combined anatomic factors predicting risk of anterior cruciate ligament injury for males and females. Am J Sports Med. 2015;43:839-847. 25583759 10.1177/0363546514563277 PMC6607022

[27] Tischer T Paul J Pape D , et al. The impact of osseous malalignment and realignment procedures in knee ligament surgery: a systematic review of the clinical evidence. Orthop J Sports Med. 2017;5:2325967117697287. 10.1177/2325967117697287 PMC5400157 28451605

[28] Todd MS Lalliss S Garcia E DeBerardino TM Cameron KL . The relationship between posterior tibial slope and anterior cruciate ligament injuries. Am J Sports Med. 2010;38:63-67. 19737987 10.1177/0363546509343198

[29] Webb JM Salmon LJ Leclerc E Pinczewski LA Roe JP . Posterior tibial slope and further anterior cruciate ligament injuries in the anterior cruciate ligament-reconstructed patient. Am J Sports Med. 2013;41:2800-2804. 24036571 10.1177/0363546513503288

[30] Wilde J Bedi A Altchek DW . Revision anterior cruciate ligament reconstruction. Sports Health. 2014;6:504-518. 25364483 10.1177/1941738113500910 PMC4212350

[31] Wordeman SC Quatman CE Kaeding CC Hewett TE . In vivo evidence for tibial plateau slope as a risk factor for anterior cruciate ligament injury: a systematic review and meta-analysis. Am J Sports Med. 2012;40:1673-1681. 22539537 10.1177/0363546512442307 PMC4168892

[32] Wright R Spindler K Huston L , et al. Revision ACL reconstruction outcomes: MOON cohort. J Knee Surg. 2011;24:289-294. 22303759 10.1055/s-0031-1292650 PMC4451059

[33] Wright RW Gill CS Chen L , et al. Outcome of revision anterior cruciate ligament reconstruction: a systematic review. J Bone Joint Surg Am. 2012;94:531-536. 22438002 10.2106/JBJS.K.00733 PMC3298683

